# The Andes Virus Nucleocapsid Protein Directs Basal Endothelial Cell Permeability by Activating RhoA

**DOI:** 10.1128/mBio.01747-16

**Published:** 2016-10-25

**Authors:** Elena E. Gorbunova, Matthew J. Simons, Irina N. Gavrilovskaya, Erich R. Mackow

**Affiliations:** a Department of Molecular Genetics and Microbiology, Stony Brook University, Stony Brook, New York, USA; b Molecular and Cell Biology Program, Stony Brook University, Stony Brook, New York, USA

## Abstract

Andes virus (ANDV) predominantly infects microvascular endothelial cells (MECs) and nonlytically causes an acute pulmonary edema termed hantavirus pulmonary syndrome (HPS). In HPS patients, virtually every pulmonary MEC is infected, MECs are enlarged, and infection results in vascular leakage and highly lethal pulmonary edema. We observed that MECs infected with the ANDV hantavirus or expressing the ANDV nucleocapsid (N) protein showed increased size and permeability by activating the Rheb and RhoA GTPases. Expression of ANDV N in MECs increased cell size by preventing tuberous sclerosis complex (TSC) repression of Rheb-mTOR-pS6K. N selectively bound the TSC2 N terminus (1 to 1403) within a complex containing TSC2/TSC1/TBC1D7, and endogenous TSC2 reciprocally coprecipitated N protein from ANDV-infected MECs. TSCs normally restrict RhoA-induced MEC permeability, and we found that ANDV infection or N protein expression constitutively activated RhoA. This suggests that the ANDV N protein alone is sufficient to activate signaling pathways that control MEC size and permeability. Further, RhoA small interfering RNA, dominant-negative RhoA(N19), and the RhoA/Rho kinase inhibitors fasudil and Y27632 dramatically reduced the permeability of ANDV-infected MECs by 80 to 90%. Fasudil also reduced the bradykinin-directed permeability of ANDV and Hantaan virus-infected MECs to control levels. These findings demonstrate that ANDV activation of RhoA causes MEC permeability and reveal a potential edemagenic mechanism for ANDV to constitutively inhibit the basal barrier integrity of infected MECs. The central importance of RhoA activation in MEC permeability further suggests therapeutically targeting RhoA, TSCs, and Rac1 as potential means of resolving capillary leakage during hantavirus infections.

## IMPORTANCE

HPS is hallmarked by acute pulmonary edema, hypoxia, respiratory distress, and the ubiquitous infection of pulmonary MECs that occurs without disrupting the endothelium. Mechanisms of MEC permeability and targets for resolving lethal pulmonary edema during HPS remain enigmatic. Our findings suggest a novel underlying mechanism of MEC dysfunction resulting from ANDV activation of the Rheb and RhoA GTPases that, respectively, control MEC size and permeability. Our studies show that inhibition of RhoA blocks ANDV-directed permeability and implicate RhoA as a potential therapeutic target for restoring capillary barrier function to the ANDV-infected endothelium. Since RhoA activation forms a downstream nexus for factors that cause capillary leakage, blocking RhoA activation is liable to restore basal capillary integrity and prevent edema amplified by tissue hypoxia and respiratory distress. Targeting the endothelium has the potential to resolve disease during symptomatic stages, when replication inhibitors lack efficacy, and to be broadly applicable to other hemorrhagic and edematous viral diseases.

## INTRODUCTION

Hantaviruses predominantly infect microvascular endothelial cells (MECs) and nonlytically cause diseases associated with increased vascular permeability (1–7). Hantavirus pulmonary syndrome (HPS) results from infection by hantaviruses present in North and South America, including Andes virus (ANDV), Sin Nombre virus (SNV), New York 1 virus, and many others (5, 8–12). However, ANDV is the only hantavirus reported to spread from person to person (5, 9–12) and to cause lethal HPS-like disease in Syrian hamsters (9, 13–15). HPS is characterized by thrombocytopenia, hypoxia, and acute pulmonary edema that leads to respiratory insufficiency and an associated 35 to 49% mortality rate (4, 7, 16, 17).

Although hantaviruses infect MECs in many organs, virtually all pulmonary MECs are reportedly infected and enlarged in HPS patients (1, 7). This unique hantavirus MEC tropism sets the stage for dysregulated MEC barrier functions to contribute to capillary leakage during HPS (1, 4, 7). The association of immune and cytokine responses with MEC permeability has been suggested (18–20), yet the same data support opposing conclusions, and steroids fail to control hantavirus disease (1, 4, 7, 21). A study of HPS in macaques indicates that pulmonary edema is observed from 6 to 13 days postinfection (dpi) without concurrent T cell or cytokine responses (22). Studies of ANDV-infected Syrian hamsters, which closely mimic human HPS (13–15), indicate that dexamethasone or cyclophosphamide treatment or depletion of macrophages or CD4^+^ or CD8^+^ T cells failed to alter the timing, onset, or severity of HPS (13, 23). In fact, immunosuppression permits SNV to cause lethal edema in Syrian hamsters (24).

Additional findings support roles for hantavirus dysregulation of infected pulmonary MECs in HPS-directed capillary permeability. Pathogenic hantaviruses engage inactive, bent α_v_β_3_ integrin conformers in order to infect MECs (25–28), and hantaviruses remain cell associated (29, 30), inhibiting α_v_β_3_ integrin-directed MEC migration days after infection (29, 31, 32). Activated α_v_β_3_ integrins normally restrict the permeabilizing effects of vascular endothelial growth factor (VEGF) by forming a complex with VEGF receptor 2 (VEGFR2) (33, 34). Pathogenic, but not nonpathogenic, hantaviruses uniquely inhibit α_v_β_3_ functions in human MECs, resulting in the hyperpermeability of MECs to VEGF or hypoxia-induced VEGF (31, 32, 35). Edema causes hypoxia, and HPS patients become acutely hypoxic, with elevated VEGF levels in pulmonary edema fluids (36). Secreted VEGF binds to endothelial cell (EC) receptors within 0.5 mm of its release (37), acting locally to disassemble adherens junctions (AJs) and induce EC permeability (34, 38). Bradykinin release following activation of the kallikrein-kinin system was also shown to increase electrical conductance, as a measure of permeability, in ANDV- and Hantaan virus (HTNV)-infected ECs (39). However, the mechanisms by which hantaviruses constitutively cause basal capillary permeability and edema that evolves into later tissue hypoxia remain to be resolved.

AJs are composed of homophilic interendothelial vascular endothelial (VE)-cadherin complexes that form the primary fluid barrier of capillaries (38, 40). Intracellularly, VE-cadherin engages the actin cytoskeleton and is dynamically regulated by extracellular and intracellular signaling pathways that control cell morphology, motility, and leukocyte extravasation (38, 40, 41). Rac1 and RhoA are cytoplasmic cellular GTPases that opposingly control the density of VE-cadherin within AJs, pore formation during diapedesis, EC barrier integrity, and capillary permeability (40, 42–46). Activation of α_v_β_3_ or focal adhesion kinase (FAK) activates Rac1, increasing the density of VE-cadherin between ECs, and FAK also engages and stabilizes actin/VE-cadherin complexes (33, 40, 47, 48). In contrast, inhibition of α_v_β_3_ prevents FAK and Rac1 activation and instead directs RhoA activation (44, 48, 49). In ECs, the conditional knockout of FAK or the RhoA inhibitor RhoGDI is sufficient to increase EC permeability and cause pulmonary edema in mice (48, 50, 51).

In HPS patients, hantavirus-infected MECs are reportedly enlarged (1, 7), providing a visible correlate of MEC dysfunction. *In vitro*, we also found that ANDV-infected MECs were enlarged (3- to 5-fold), with hypoxia increasing both the number of enlarged infected MECs and MEC permeability (52, 53). In contrast, infection of MECs with nonpathogenic Tula virus (TULV) or mock infection resulted in 2 to 10% enlarged MECs under hypoxic conditions and failed to enhance MEC permeability (31, 32, 53). Cell size is controlled by mTOR-directed phosphorylation of S6 kinase (S6K) (54) and normally inhibited by TSC repression of the mTOR GTPase Rheb (54, 55). ANDV-induced increased MEC size was directed by activating the Rheb-mTOR-pS6K signaling pathway (53). Tuberous sclerosis complexes (TSCs) normally inhibit Rheb-directed mTOR activation (54, 56), and mutations in TSC proteins (TSC1-hamartin, TSC2-tuberin) constitutively activate Rheb-mTOR-pS6K and increase cell size (54, 56). Intriguingly, TSCs also regulate Rac1 and RhoA GTPases that play fundamental antagonistic roles in the control of EC permeability (40, 45, 57–59). This suggested that ANDV regulation of TSCs may increase both MEC size and capillary leakage in HPS.

In this study, we evaluated ANDV infection and N protein regulation of TSCs that result in Rheb and RhoA activation in MECs. Our results indicate that expression of the ANDV N protein alone in MECs increases cell size and activates Rheb-mTOR-pS6K by binding to TSCs. Our studies revealed that ANDV N protein coprecipitates TSC2, assembled TSC complexes, and the TSC inhibitor 14-3-3 (60–62). Consistent with this, we found that ANDV infection or N protein expression in MECs activated RhoA and reduced levels of the RhoA inhibitor p190RhoGAP and the Rac1 activator TIAM1. Small interfering RNA (siRNA) knockdown of RhoA, expression of dominant-negative RhoA, or inhibition of RhoA/ROCK with fasudil or Y27632 was found to reduce ANDV-directed MEC permeability by 80 to 90%. These findings demonstrate that ANDV activation of RhoA causes MEC permeability and suggest an underlying edemagenic mechanism that may constitutively decrease the barrier integrity of ANDV-infected MECs. These findings implicate RhoA, TSCs, and Rac1 as potential therapeutic targets for resolving capillary leakage during ANDV infection and a potential means of resolving edema during symptomatic HPS stages.

## RESULTS

### ANDV N protein expression in human endothelial cells increases cell size.

**FIG 1:**
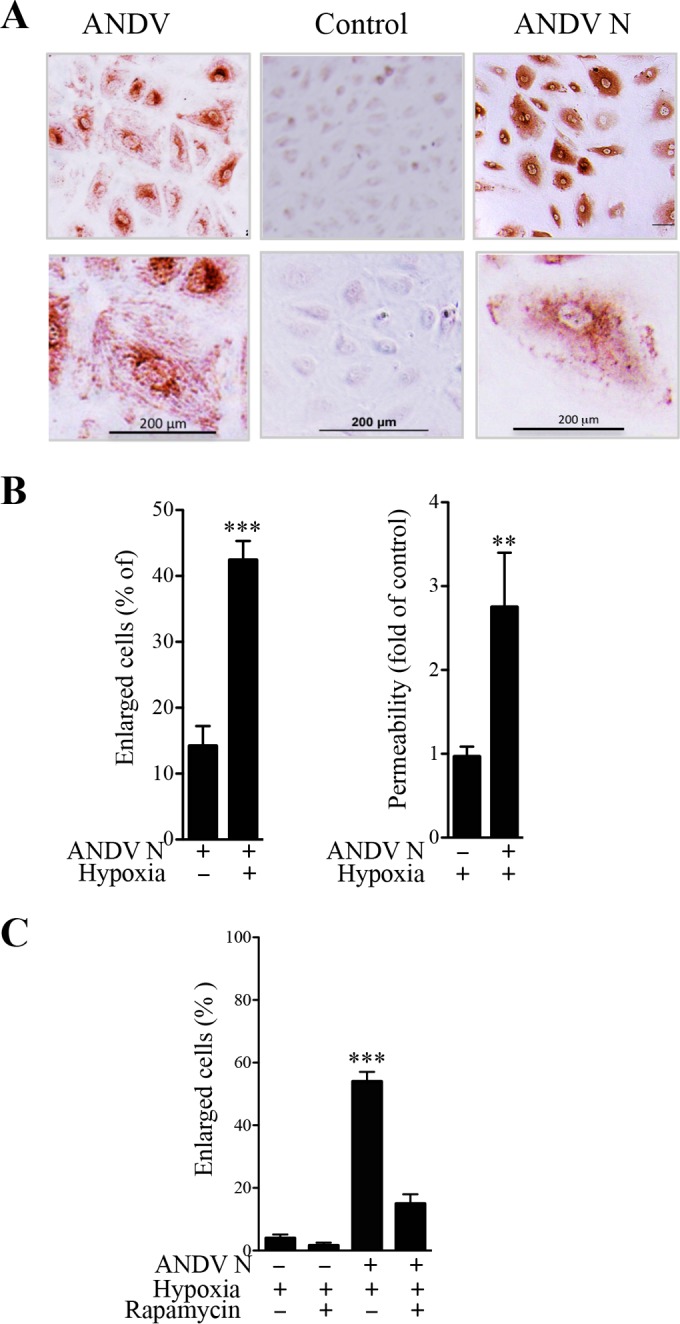
ANDV N protein expression induces mTOR-directed endothelial cell enlargement. (A) Pulmonary MECs were infected with ANDV or transduced with a lentivirus expressing ANDV N protein and puromycin selected (113). MECs were immunoperoxidase stained for N protein and visualized by light microscopy. (B) The percentage of enlarged MECs expressing ANDV N under normoxic (20% O_2_) or hypoxic (1% O_2_) conditions was measured and quantified with NIH Image (31, 32, 53). The permeability of N-protein-expressing MECs relative to that of control MECs was determined by Transwell permeability to FITC-dextran (40 kDa) incubated under normoxic or hypoxic conditions (31, 32, 53). (C) Control and ANDV N-protein-expressing MECs were assayed for the effect of VEGF (20 ng/ml; 1 h) on enlarged cells (≥3× normal size) in the presence or absence of rapamycin (53). Data represent results of three independent experiments (**, *P* < 0.01; ***, *P* < 0.001).

The mechanism by which ANDV activates mTOR, increases MEC size, and causes MEC permeability remains to be defined. Hantavirus N proteins are highly expressed during infection (63, 64), yet roles for hantavirus proteins in MEC dysfunction and permeability have not been studied. Here we analyzed the constitutive expression of ANDV N protein in early-passage primary human pulmonary MECs. MECs were lentivirus transduced to express ANDV N protein and puromycin selected. ECs persistently expressed N protein in >95% of MECs without notable effects on cell viability or loss of N protein expression in the absence of puromycin selection (Fig. 1A). Similar to ANDV infection (53), we noted that ~15% of N-protein-expressing MECs were enlarged (three to five times normal size) (Fig. 1A and B). Hypoxic conditions increased the number of enlarged MECs (40 to 50%) and the permeability of N-protein-expressing MECs (~3-fold) (Fig. 1B). In comparison, ~5% of mock-transduced, hypoxia-treated control MECs were enlarged (Fig. 1C) (53). Under hypoxic conditions, the percentage of enlarged N-protein-expressing MECs was dramatically reduced by addition of the mTOR inhibitor rapamycin (Fig. 1C).

### ANDV N protein induces mTOR-directed phosphorylation of S6K.

**FIG 2:**
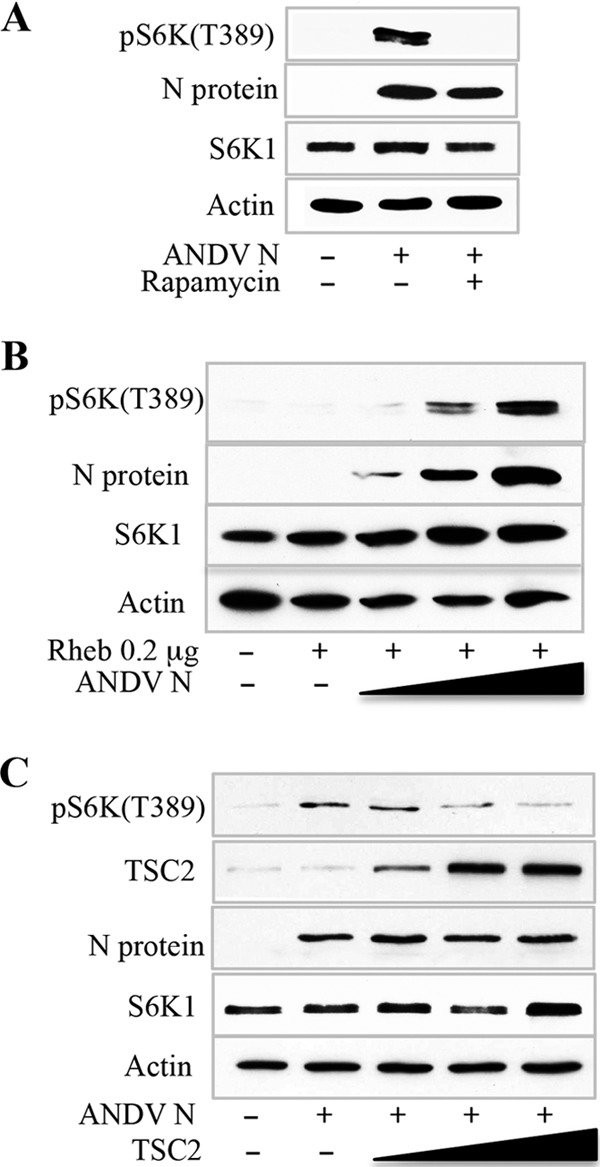
ANDV N-protein-induced mTOR phosphorylation of S6K is TSC2 sensitive. TSC repression of Rheb-mTOR-pS6K restricts cell size (54). (A) MECs transduced and puromycin selected to persistently express ANDV N protein or mock transduced were evaluated for mTOR-directed phosphorylation of S6K under hypoxic conditions (53). Control or N-protein-expressing MECs were assayed for changes in S6K phosphorylation by WB assay with a phosphospecific antibody to S6K (T389) (cell signaling) in the presence or absence of rapamycin (20 ng/ml) as previously described (53). (B) HEK293T cells were cotransfected with increasing amounts of a plasmid expressing the N protein and a constant amount of Rheb and assayed for pS6K, total S6K, and actin by WB (53). (C) ANDV N-protein-directed phosphorylation of S6K was assayed in the presence of increasing amounts of a plasmid expressing TSC2. Lysates were assayed by WB assay for pS6K, total S6K, TSC2, N protein, and actin levels.

TSCs regulate cell size by inhibiting the mTOR-specific GTPase Rheb (54, 55). Mutations in the TSC1 or TSC2 protein result in increased cell size by derepressing Rheb and constitutively activating mTOR-directed phosphorylation of S6K (54). Analysis of N-protein-transduced MECs revealed that N protein expression directed the phosphorylation of S6K under hypoxic conditions (Fig. 2A). In contrast, S6K was not phosphorylated by hypoxia treatment of MECs alone (Fig. 2A) and pS6K responses of N-expressing MECs was blocked by rapamycin (Fig. 2A). Consistent with this, expression of N protein in HEK293 cells in the presence of Rheb dose dependently increased S6K phosphorylation, while expression of Rheb alone failed to increase pS6K (Fig. 2B). Interestingly, expression of increasing amounts of TSC2 resulted in a concomitant decrease in N-protein-directed S6K phosphorylation (Fig. 2C), suggesting that N-directed mTOR activation is TSC2 mediated. Collectively, these findings indicate that ANDV N protein increases the size of MECs by activating the Rheb-mTOR-pS6K signaling pathway.

### ANDV N protein binds TSCs via interactions with N-terminal domains of TSC2.

**FIG 3:**
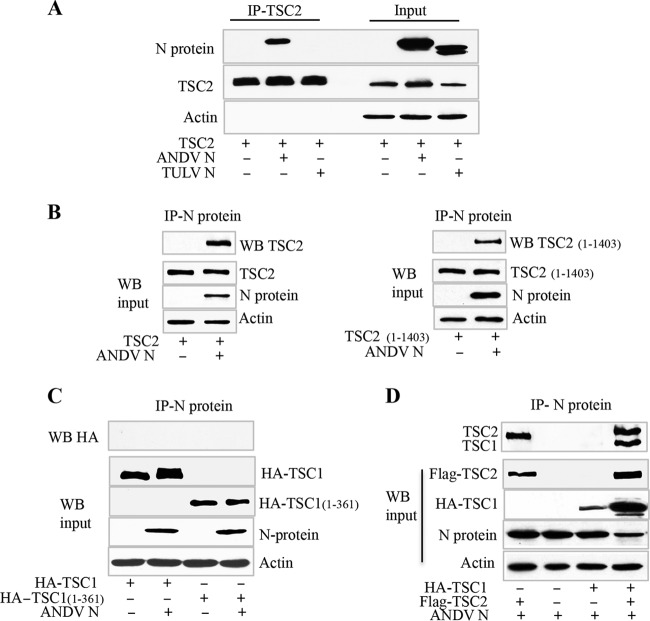
ANDV N protein coimmunoprecipitates TSC2 via N-terminal domains. (A) HEK293T cells were cotransfected with plasmids expressing the ANDV or TULV N protein and hemagglutinin (HA)-tagged TSC2. Cell lysates (114) were immunoprecipitated with anti-HA antibody, evaluated for immunoprecipitated TSC2 protein and coprecipitated N proteins by WB (left), and simultaneously analyzed for input protein by WB of N protein, TSC2, and actin (right) (114, 115). (B) HEK293 cells transfected with plasmids expressing N protein, Flag-tagged TSC2, or a C-terminal truncation of TSC2 containing residues 1 to 1403 were immunoprecipitated (IP) with anti-N rabbit antibody and assayed by WB for coprecipitated TSC2 or truncated TSC2, as well as for input protein, by direct WB analysis of lysates for TSC2, N protein, and actin (116). (C) HEK293T cells were cotransfected with plasmids expressing ANDV N protein, HA-tagged TSC1, or an HA-tagged C-terminal truncation of TSC1 containing residues 1 to 361. Cell lysates were immunoprecipitated with antibody to ANDV N protein and assayed for coprecipitation of TSC1 or TSC1 truncations by WB (114) or assayed directly for input protein by WB for HA-TSC1, N protein, and actin (114, 115). (D) HEK293 cells were cotransfected with the TSC2-, HA-TSC1-, and N-protein-expressing plasmids indicated and assayed for input protein by WB and for N protein coprecipitation of TSC2 and TSC1 by WB.

The findings described above suggest that N protein may alter normal TSC repression of Rheb. TSC1 and TSC2 form a complex that inhibits Rheb-directed mTOR activation through a GTPase-activating protein (GAP) domain in the TSC2 C terminus (55). We previously reported that ANDV, but not nonpathogenic TULV, activates mTOR-pS6K and increases cell size (53). In order to determine if the TULV and ANDV N proteins differ in the ability to interact with TSCs, we coexpressed TSC2 with ANDV or TULV N protein and assayed N protein interactions with TSC2. We immunoprecipitated TSC2 from cell lysates and found that TSC2 selectively coprecipitated ANDV, but not TULV, N protein (Fig. 3A). These findings are consistent with ANDV activation of mTOR and prompted the evaluation of ANDV N protein interactions with additional TSC components that normally repress Rheb (56, 58). HEK293 cells were transfected with plasmids expressing ANDV N, TSC1, TSC2, or truncated TSC proteins and reciprocally evaluated for coimmunoprecipitation by ANDV N protein. ANDV N protein coprecipitated TSC2 and a C-terminal TSC2 truncation (1 to 1403) lacking the GAP domain (Fig. 3B). In contrast, N protein failed to coprecipitate TSC1 or a C-terminal TSC1 truncation (1 to 361) (Fig. 3C). In cells coexpressing TSC1 and TSC2, we found that N protein coprecipitated both TSC2 and TSC1 (Fig. 3D). These findings suggest that ANDV N interacts with assembled TSC1-TSC2 complexes through interactions with the N terminus of TSC2 that are independent of the TSC2 GAP domain.

### ANDV N protein binds TSCs in the presence or absence of the TSC inhibitor 14-3-3.

**FIG 4:**
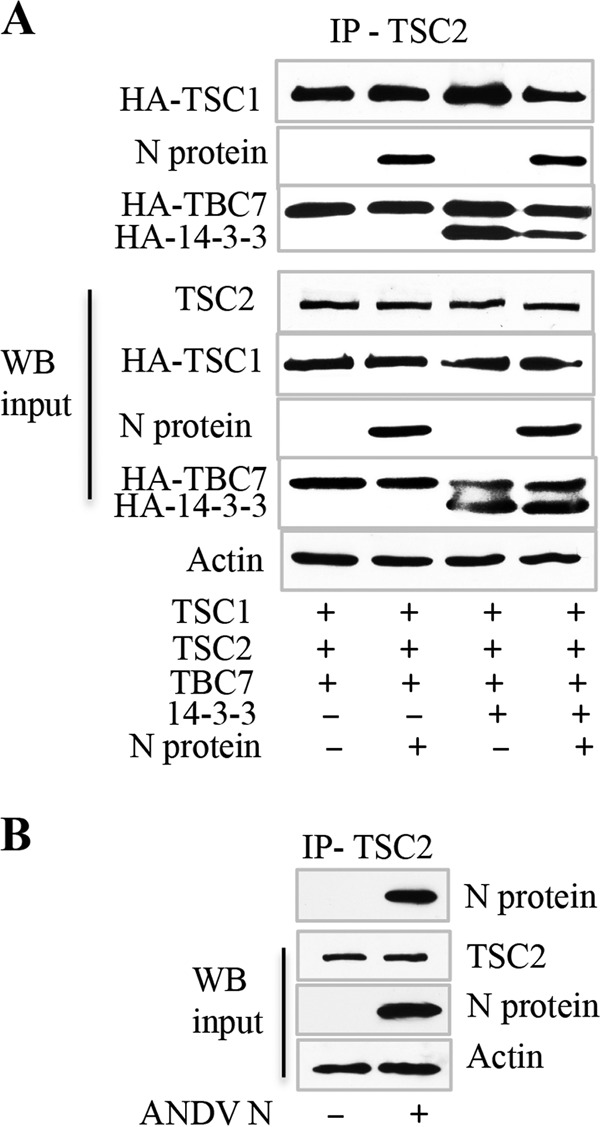
TSC2 coimmunoprecipitates ANDV N protein in assembled TSC complexes from ANDV-infected MECs. (A) HEK293 cells were cotransfected with plasmids expressing TSC2, N protein, HA-TSC1, HA-TBC1D7, and HA–14-3-3 as indicated. Cell lysates were immunoprecipitated (IP) with antibody to TSC2 and assayed for coprecipitation of N protein and TSC1, TBC1D7, and the TSC inhibitor 14-3-3 as indicated. Lysates were analyzed for individually expressed input proteins and actin by WB. (B) MECs were ANDV infected at an MOI of 0.5, and at 3 dpi, cell lysates were immunoprecipitated with antibody to endogenous TSC2 and assayed for coprecipitation of ANDV N protein by WB. WB of endogenous TSC2 and ANDV N protein in infected MEC lysates was analyzed for total input TSC2, N protein, and actin levels.

In addition to TSC1 and TSC2 components, TSCs are present as ternary complexes containing TBC1D7 (62, 65), and TSC regulation of Rheb is inhibited by recruitment of the scaffold protein 14-3-3 (60, 61). Here we immunoprecipitated TSC2 and analyzed N protein interactions with TSCs containing TBC1D7 and 14-3-3. We found that TBC1D7, TSC1, and N protein were coprecipitated by TSC2 and that N protein formed a complex with TSCs in the presence or absence of 14-3-3 (Fig. 4A). These findings suggest that, instead of disrupting TSCs, N protein binding to TSC2 mediates its association with assembled TSCs and that N protein binding to TSCs is discrete from the binding of inhibitory 14-3-3 proteins (Fig. 4A) (60–62).

We further evaluated endogenous TSC2 interactions with N protein following ANDV infection of MECs. We found that immunoprecipitation of endogenous TSC2 from ANDV-infected MECs resulted in coprecipitation of the ANDV N protein (Fig. 4B). These findings validate coexpression studies by demonstrating endogenous interactions of the Rheb inhibitor TSC2 with N protein during ANDV infection. Together, these findings indicate that ANDV N protein binding to TSCs and TSC–14-3-3 complexes prevents TSC repression of Rheb and results in the activation of mTOR-pS6K signaling pathways.

### ANDV infection and N protein expression activate RhoA in MECs.

**FIG 5:**
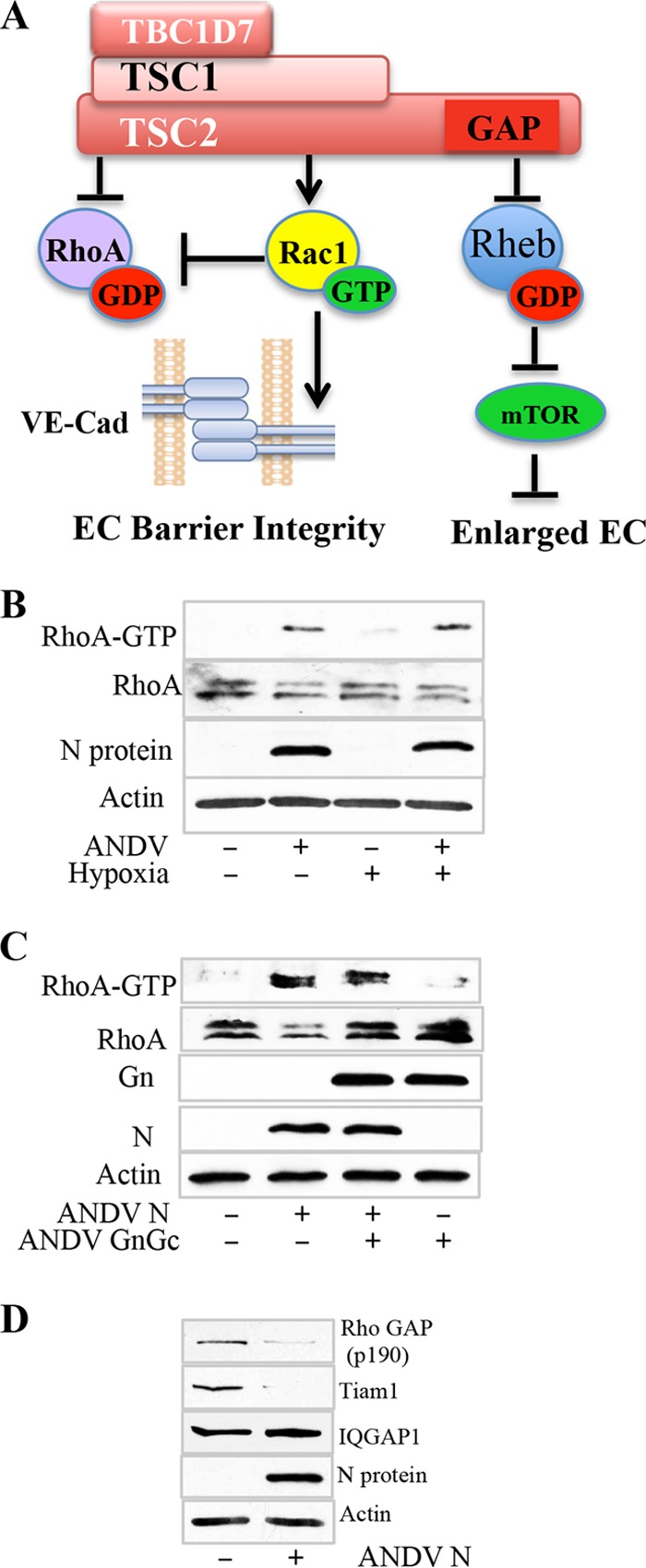
ANDV and ANDV N protein activate RhoA. (A) TSCs normally inhibit Rheb and RhoA and enhance Rac1-directed EC barrier integrity. N protein derepression of TSC-Rheb suggests that N may similarly direct RhoA activation and MEC permeability. (B) ANDV-infected MECs were assayed for RhoA activation (Rho-GTP) with the GST-Rhotekin-RBD assay (Cytoskeleton Inc.) and for total RhoA and N protein levels under hypoxic (Fig. 1) or normoxic conditions. (C) Control MECs and MECs transduced to express ANDV N protein, N, and GnGc or GnGc alone were assayed for RhoA activation as described above and for RhoA and ANDV-expressed proteins. (D) Control MECs or MECs constitutively expressing ANDV N protein were assayed for total p190RhoGAP, Rac1 GEF, and TIAM1 protein levels and for IQGAP, N protein, and actin by WB.

Collectively, our findings suggest that N protein binds TSCs and inhibits TSC repression of Rheb. However, TSCs also regulate signaling responses directed by Rac1 and RhoA GTPases (57–59, 66) that antagonistically regulate EC permeability (Fig. 5A) (40, 43, 50). A wide range of factors activate RhoA to cause EC permeability (38, 42, 49, 51, 67), and this prompted us to determine if RhoA was activated by ANDV infection of MECs (40, 46). We assayed activated (GTP-bound) RhoA by using Rhotekin binding domain assays and found that ANDV infection of MECs constitutively activated RhoA and that RhoA activation was independent of hypoxic conditions (Fig. 5B). We similarly analyzed MECs expressing ANDV N protein and found that RhoA was constitutively activated (Fig. 5C). In contrast, lentivirus expression of GnGc alone in MECs did not activate RhoA and coexpression of N and GnGc in MECs resulted in RhoA activation similarly to N protein expression alone (Fig. 5C). These findings indicate that RhoA activation is uniquely directed by the ANDV N protein and that ANDV infection directs the basal activation of RhoA in human MECs independently of hypoxia. Since RhoA activation is a prominent cause of MEC and capillary permeability (43, 46, 50, 51, 68), our results suggest that RhoA activation by ANDV is an underlying mechanism of diminished MEC barrier integrity and basal capillary leakage.

GTPase-specific GAPs and GEFs (guanine nucleotide exchange factors), respectively, inhibit or activate Rac1 and RhoA (50, 69–71). We analyzed N-protein-expressing MECs for changes in p190RhoGAP, TIAM1, and IQGAP, which are respective regulators of RhoA, Rac1, and cdc42 GTPases. We found that MECs expressing the ANDV N protein had dramatically reduced levels of the RhoA inhibitor p190RhoGAP and the Rac1 activator TIAM1, while levels of IQGAP remained unchanged in N-protein-expressing cells (Fig. 5D). These findings are consistent with the idea that N protein expression prevents p190RhoGAP repression of RhoA. Since TSCs normally inhibit RhoA and activate Rac1, our findings are consistent with N protein activation of RhoA by coordinated inhibition of TSCs, GAPs, and GEFs, which determine the balance of Rac1 and RhoA activation and MEC barrier integrity (70, 71). However, it remains to be determined if these changes are a cause of RhoA activation or whether additional factors that control RhoA (i.e., RhoGDI, FAK, Syx, Vav2, and p115RhoGEF) (48–50, 69, 71) are engaged by ANDV infection or N expression to constitutively activate RhoA.

### ANDV-induced MEC permeability is blocked by inhibition of RhoA.

**FIG 6:**
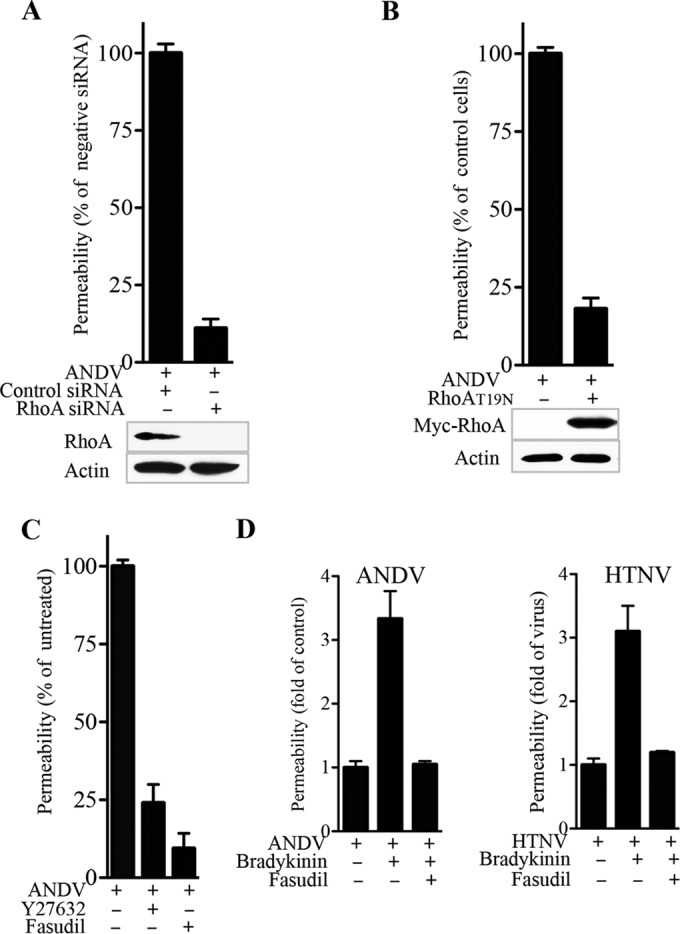
RhoA inhibitors block ANDV-directed MEC permeability. (A) MECs were ANDV infected (MOI, 1) and transfected at 2 dpi with control siRNA or siRNA to RhoA. RhoA and actin protein levels were assayed by WB, and MEC permeability was assayed as described in the legend to Fig. 1B by Transwell assay, and results are presented as a percentages of control ANDV-directed MEC permeability (31, 32). (B) MECs were transduced with a lentivirus expressing dominant-negative RhoA(T19N) (73) and subsequently ANDV infected and assayed as described above for MEC permeability directed by ANDV infection. (C) MECs were ANDV infected, and at 3 dpi, the RhoA inhibitor fasudil or Y27632 (10 µM) was added to cells 6 h prior to analysis of MEC permeability. Monolayer permeability was assayed as described for Transwell monolayer permeability to FITC-dextran (40 kDa) for 30 min with FITC in the lower chambers quantitated by fluorimetry. (D) MECs were mock, ANDV, or HTNV infected (MOI, 1) in Transwell plates in triplicate, and at 3 dpi, MECs were incubated in EBM–0.5% BSA for 2 h. MECs were treated with or without fasudil (10 µM) for 1 h and subsequently stimulated with bradykinin (1 µM) in the presence of FITC-dextran (40 kDa) for 30 min prior to fluorimetric quantitation of medium in the lower chambers and comparison to mock-infected controls. Data are representative of results from three independent experiments.

Activation of RhoA directs actin contraction and the disassembly of VE-cadherin within AJs that controls capillary permeability (38, 41, 43, 46, 50, 70). We previously reported that ANDV and hemorrhagic fever with renal syndrome (HFRS)-causing HTNV, but not TULV, infections of MECs induce VE-cadherin disassembly and enhance MEC permeability in response to hypoxia or VEGF (31, 32, 35, 72). The findings described above suggest that RhoA activation may be an underlying edemagenic mechanism that causes capillary leakage and basal pulmonary edema during ANDV infection. Here we determined if ANDV-induced permeability is RhoA mediated by analyzing responses of MECs to discrete RhoA inhibitors. We transfected MECs with RhoA siRNAs or transduced MECs to express dominant-negative RhoA(T19N) (73) and found that RhoA expression levels were specifically reduced in siRNA-treated MECs and increased in RhoA(T19N)-expressing cells (Fig. 6A and B). Using a gold standard fluorescein isothiocyanate (FITC) Transwell permeability assay (31, 32, 53), we found that RhoA-specific siRNA resulted in a 90% reduction of ANDV-induced permeability (Fig. 6A). Similarly, transduction of MECs with a lentivirus expressing an inactive RhoA(T19N) mutant protein resulted in an 80% reduction in ANDV-induced MEC permeability (Fig. 6B).

We further analyzed the effects of the RhoA and Rho kinase (ROCK) inhibitors fasudil (HA-1077) and Y27632 (70, 74) for the ability to reduce ANDV-induced EC permeability. At 3 days after ANDV infection, we added RhoA inhibitors to cells 6 h prior to analysis of MEC permeability. We found that the addition of fasudil or Y27632 dramatically reduced ANDV-induced MEC permeability 80 to 90% (Fig. 6C). A prior study showed that ANDV and HTNV enhanced bradykinin-directed permeability (39). As a result, we determined whether ANDV- and HTNV-directed MEC permeability responses induced by bradykinin were inhibited by the ROCK inhibitor fasudil. We found that the addition of bradykinin to ANDV- and HTNV-infected MECs increased permeability ~3-fold and that coadministration of fasudil inhibited permeability to control levels (Fig. 6D).

**FIG 7:**
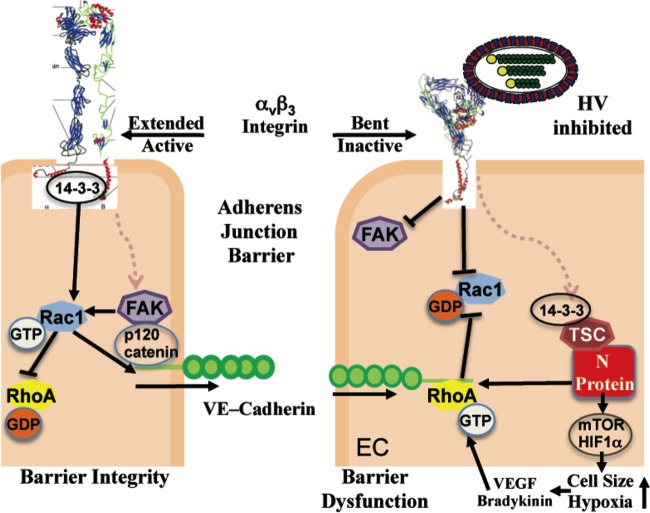
Fundamental role of RhoA activation in ANDV-induced permeability. A potential model of RhoA activation directed by ANDV infection of endothelial cells is shown. Rac1 activation, which normally enhances VE-cadherin assembly within AJs and maintains MEC barrier integrity (40, 46, 58, 86, 87), is depicted on the left. Potential changes in RhoA and Rac1 responses following ANDV infection of MECs are presented on the right. RhoA activation by N protein may be exacerbated by additional responses to ANDV infection that dysregulate normal α_v_β_3_ integrin and FAK responses and are enhanced by hypoxia and mTOR-directed increases in HIF1α. Extracellularly, pathogenic hantaviruses bind bent, inactive α_v_β_3_ integrins (27), blocking integrin and associated FAK and Rac1 responses that stabilize AJs (48, 79, 117). Intracellularly, ANDV N protein binds and inhibits TSCs that normally repress RhoA (58, 59, 86)- and Rheb-mTOR (56)-directed changes in cell size (54, 82), HIF1α induction (60), and permeability (43, 48, 50, 68). N protein activates RhoA, which constitutively increases MEC permeability and also explains ANDV-enhanced MEC permeability in response to hypoxia (52, 53), bradykinin (39), and VEGF (31, 32, 36, 91), which further activate RhoA (49, 68). These findings are consistent with ANDV activation of RhoA, hypoxia-enhanced MEC permeability, and the ability of RhoA inhibitors to block this common downstream permeability nexus (49).

These findings indicate that ANDV-induced MEC permeability is RhoA directed and blocked by inhibition of RhoA activation. Collectively, our findings suggest a mechanism by which ANDV induces basal changes in MEC permeability and cell size through N protein interactions with TSC that derepress Rheb and RhoA GTPases. Our findings suggest the potential for RhoA to be a conserved downstream target for hantavirus therapeutics, which may reduce or resolve basal ANDV-induced edema by inhibiting RhoA activation or activating pathways that restore Rac1 activation and TSC function (Fig. 7).

## DISCUSSION

ECs contain unique receptors, junctions, and signaling pathway effectors that regulate immune cell and platelet binding and activation, transcytosis, vascular tone, and the activation of complement and clotting cascades that collectively regulate hemostasis (75). ECs regulate vascular barrier functions through a series of failsafe mechanisms that are in place to prevent a lethal breach of barrier integrity (33, 34, 38, 75, 76). As a result, it is likely that several EC functions need to be inhibited to cause hemorrhagic or edematous diseases.

ECs are the primary cellular targets of hantavirus infection (1, 6, 7), and this focuses studies of pathogenesis on mechanisms by which hantaviruses dysregulate MEC functions (31, 72, 77) in order to increase vascular permeability and cause the diseases HPS and HFRS (2, 3, 16). Mutation or knocking out of β_3_ integrins causes vascular leakage (33, 78), and pathogenic hantaviruses bind and inhibit the function of β_3_ integrins present on platelets and ECs (25–28). HPS patients are acutely thrombocytopenic (7), and on MECs, α_v_β_3_ integrins play a fundamental role in cell migration, the formation of focal adhesions, Rac1 activation, and the regulation of VEGFR2-directed permeability (48, 79). Hypoxia, observed at late stages of HPS (4, 7), induces the permeability factor VEGF (34, 53), as well as bradykinin receptors that direct permeability in response to activation of the kallikrein-kinin system (39, 67, 80, 81). In fact, VEGF levels are increased in HPS pulmonary edema fluids (31, 32, 36), and activation of the kallikrein system in HV-infected cells releases bradykinin and increases EC permeability (39). Capillary permeability is commonly mediated by downstream RhoA activation, and findings presented here demonstrate that the ANDV N protein activates RhoA.

In HPS patients, nearly every pulmonary MEC is infected and enlarged (1, 7); similarly, ANDV infection of MECs *in vitro* results in the generation of enlarged cells (52, 53). Here we show that expression of the ANDV N protein in MECs is sufficient to cause MEC enlargement and activate Rheb-mTOR-pS6K and RhoA signaling responses. TSCs normally repress Rheb (56, 82), and expression of ANDV N protein dose dependently increased S6K phosphorylation that was inhibited by expression of TSC2. We observed that ANDV N protein binds to endogenous or expressed TSC2 and that, instead of displacing TSC components, N bound to an assembled TSC complex with or without the TSC inhibitor 14-3-3 (60, 62, 83, 84). This suggests a novel mechanism by which ANDV N protein inactivates TSCs to control cell size and potentially enhance mTOR-directed increases in HIF1α that may contribute to hypoxia-induced responses of ANDV-infected MECs (60, 62).

TSCs also control the activity of Rac1 and RhoA GTPases that antagonistically control barrier integrity and capillary permeability (42, 46, 51, 58, 60, 85, 86). RhoA activation directs stress fiber organization and contraction, inhibits Rac1 activation, impairs VE-cadherin assembly, and increases vascular permeability (43, 48, 50). In contrast, Rac1 activation directs the formation of filopodia, increases the assembly of VE-cadherin homodimers between MECs, increases capillary barrier integrity, and inhibits RhoA (40, 46, 58, 86, 87). Thus, the balance between the activation of Rac1 and that of RhoA critically regulates AJ barrier function and vascular permeability (38, 40, 41, 70). We found that ANDV infection or N protein expression in MECs constitutively activates RhoA. This suggests a program by which the ANDV N protein inhibits TSC regulation, activates RhoA, and tips the balance from MEC integrity to one of basal MEC leakage (Fig. 7).

Correlates of basal EC permeability during HV infection have not previously been found, in part because vessels, but not EC monolayers, are under pressure and even small changes in barrier integrity are exacerbated in capillaries. In fact even inapparent cellular stresses like breathing-directed cyclic stretching of the pulmonary endothelium (88, 89) may contribute to capillary leakage when uncoupled from normal MEC integrity. The cause of vascular leakage during hantavirus diseases has been speculated to stem from a wide range of effectors, including growth factors, kinins, immune responses, cytokines, T cells, and permeability factors (18–20, 29, 31, 32, 35, 36, 39, 77, 90). Although several factors are likely to contribute to permeability, immunosuppression of HV patients has no effect on the disease (21) and recent findings suggest that immune responses are not determinants of vascular leakage in animal models of ANDV infection (13, 22, 23). Prior studies have shown that ECs infected by pathogenic, but not nonpathogenic, hantaviruses are hyperpermeabilized by VEGF addition or by hypoxic conditions observed at late stages of HPS (31, 32, 35, 53, 91, 92). In addition, HV-infected ECs are hyperresponsive to bradykinin-directed EC permeability (39). Hypoxia or VEGF addition directed the nondegradative internalization of VE-cadherin within HV-infected MECs (31, 32, 35, 53), although another study suggested that VE-cadherin was transiently degraded after VEGF addition (91).

Activated RhoA is linked to EC permeability directed by thrombin, tumor necrosis factor alpha, and histamine, as well as bradykinin and VEGF (38, 42, 49, 67, 80, 85). Findings presented here demonstrate a role for RhoA activation in MEC permeability during ANDV infection (Fig. 6A to C) and also show that inhibition of RhoA blocks bradykinin-directed permeability in ANDV and HTNV-infected ECs (Fig. 6D). This implicates RhoA activation as a cause of basal changes in MEC integrity that contribute to vascular leakage and edema (31, 32, 36, 39). However, hypoxic conditions also induce bradykinin receptors and VEGF (46, 67, 93), and this further suggests a mechanism for ANDV to amplify RhoA-directed permeability under hypoxic conditions (38, 41, 43, 46, 49, 60). Given the fundamental role of RhoA activation in basal and hypoxia-directed EC permeability (42, 43), these findings suggest RhoA as a central downstream target of edema during HPS.

Additional MEC functions dysregulated by hantavirus infection may also exacerbate N-protein-directed RhoA activation. Both α_v_β_3_ integrins and FAK normally activate Rac1 (27, 48, 79), yet pathogenic hantaviruses block α_v_β_3_ integrin and FAK activation during infection (27, 29, 30). This suggests a role for ANDV inhibition of extracellular α_v_β_3_ integrin responses as a means of reducing Rac1-directed barrier integrity and enhancing RhoA activation during ANDV infection. Another potential way for β_3_ integrins and RhoA to contribute to pulmonary edema is provided by neutrophil recruitment to pulmonary compartments during HPS (7, 94–96). As neutrophils traverse the endothelium to enter tissues, pores are formed in ECs and pore assembly and closure are regulated by Rac1, RhoA, and β_3_ integrins (44, 95, 96). In ANDV-infected MECs, inhibition of β_3_ and Rac1 and activation of RhoA may increase the duration of pore opening and thereby diapedesis alone may trigger pulmonary edema in HPS patients. As a result, activating α_v_β_3_ and Rac1 may be investigated as synergistic targets for enhancement of EC barrier function and for inhibition of RhoA activation (Fig. 7). Whether extracellular integrin blockade (48, 79, 97) or neutrophil extravasation contributes to RhoA activation and ANDV-directed MEC permeability remains to be investigated.

There are currently no therapeutic approaches for treating hantavirus-induced diseases or reducing lethal outcomes of HPS infections (21). Interferon and replication inhibitors are efficacious prophylactically but not in viremic or symptomatic patients (5, 21, 98). However, one Puumala virus patient recovered after being given a dose of the bradykinin antagonist icatibant (90) and this supports a role for bradykinin in HFRS pathogenesis (39). However, further studies are needed to determine if icatibant or several additional therapeutics provided to the patient played a key role in recovery (90).

Our findings provide a mechanism for basal capillary permeability during ANDV infection of MECs and uniquely reveal RhoA as a potential therapeutic target for restoring MEC integrity and resolving HPS (43, 49, 80, 94). Since RhoA is a central downstream signaling effector (42, 43, 46, 68, 94, 99, 100), blocking of RhoA activation may commonly inhibit constitutive and hypoxia-directed EC permeability responses that are dysregulated by ANDV infection. ANDV-directed permeability was dramatically reduced by the pharmacological RhoA/ROCK inhibitors fasudil and Y27632 (74, 101, 102), and the approval of fasudil for use in humans (102, 103) suggests its immediate therapeutic potential. Findings presented here rationalize studying these and other RhoA inhibitors for their efficacy in resolving lethal HPS disease in a biosafety level 4 (BSL4) Syrian hamster model (15, 24).

On the basis of our findings, additional inhibitors that protect endothelial barrier function by activating Rac1 and TSCs or indirectly impact Rac1/RhoA also have the potential to inhibit capillary leakage and therapeutically resolve or reduce HPS disease. Prostaglandin E2 promotes Rac1 activation, and forskolin and rolipram protect EC barrier function by activating TSCs and preventing Rac1 inhibition (104, 105). Statins were previously noted to stabilize the endothelium by targeting 3-hydroxy-3-methylglutaryl-coenzyme A reductase, resulting in reduced RhoA geranylgeranylation required for RhoA activation (99, 106, 107). Activation of α_v_β_3_ integrins (108) or use of compounds that lead to Rac1 activation (i.e., SEW2871 [109], angiopoietin 1 [87, 110], and FTY720 [31, 111]) may similarly inhibit edemagenic RhoA-directed responses of ANDV-infected ECs. Although the responses described here were studied in an ANDV-specific context, they appear to be applicable to HFRS-causing HTNV (Fig. 6D), and the ubiquitous role of RhoA in vascular permeability (38, 42, 46, 49, 51, 67, 81) suggests that this approach may be germane to other hemorrhagic and edematous viruses.

## MATERIALS AND METHODS

### Cells and virus.

VeroE6 (ATCC CRL 1586) and HEK239T (ATCC CRL 1573) cells were grown in Dulbecco’s modified Eagle’s medium, 10% fetal calf serum, and antibiotics as previously described (31). Human pulmonary MECs were purchased from Cambrex Inc., grown in endothelial growth medium 2MV (Lonza), and supplemented as previously described (31). ANDV (CHI-7913) was cultivated in BSL3 facilities (31). Viral titers were determined in VeroE6 cells, MECs were ANDV infected at a multiplicity of infection (MOI) of 0.5 or mock infected, and cells were >90% infected at 3 dpi, as determined by focus assay of infected MECs with anti-N-protein antibodies and immunoperoxidase staining with 3-amino-9-ethylcarbazole (25, 26). MECs infected with pathogenic ANDV (MOI, 0.5) or persistently expressing ANDV N protein were incubated for 18 h under hypoxic conditions (1% O_2_ by N_2_ displacement, 5% CO_2_ in a multigas incubator [MCO-19M Sanyo Scientific], or cobalt-chloride [100 µM] treated [Sigma]) to induce hypoxia in basal EBM-2 with 0.5% bovine serum albumin (BSA) for 6 h (52, 53). Cells more than three times normal MEC size were considered to be enlarged and were quantitated by microscopy (10 fields, 1,500 cells in duplicate wells) with NIH Image.

### Plasmids and constructs.

Plasmids expressing TSC2, TSC1, TBC1D7, 14-3-3, S6K, and Rheb were obtained from Addgene (14129, 19911, 32047, 13270, 26610, and 19996). ANDV nucleocapsid open reading frames were PCR amplified and inserted into the pLenti-CMV-GFP-Puro vector at the BamHI and XbaI sites, and HEK293T cells were cotransfected with third-generation lentiviral packaging plasmids p-RSV-Rev, pMD.2G, and pMDLg/pRRE (Addgene 658-5, 12259, 12251, and 12253) (112, 113) to generate lentivirus expressing the ANDV N protein. RhoA(T19N) (73) was subcloned from the pRK5myc RhoA-T19N plasmid into the pLenti-GFP-hygro vector at the BamHI and XbaI sites (Addgene 12967, 15901, 17446, and 93425) and used as described above to generate lentiviruses for MEC transduction. Passage 3 MECs were transduced with recombinant ANDV N lentiviruses at an MOI of 5, initially puromycin selected (0.3 µg/ml), and passed in the absence of puromycin prior to studies at passages 6 and 7. ANDV N protein expression was detected in >95% of the transduced MECs. RhoA(T19N) lentiviruses were generated as described above and assayed for RhoA expression by Western blotting (WB). HEK cells were transfected with plasmids by using calcium phosphate, and siRNAs were purchased from SA-Biosciences and transfected into ECs with Surefect as previously described (32).

### Antibodies and inhibitors.

Antibodies to RhoA, S6K, TSC2, p190RhoGAP, TIAM1, IQGAP, HA-Tag, and Myc-Tag were purchased from Santa Cruz and antibodies to actin, Flag, and Phospho-70S6K were from Life Sciences. Anti-ANDV Gn monoclonal antibody was purchased from United States Biologicals. Anti-N-protein polyclonal rabbit sera made to NY-1V N protein was previously described (25, 26), and RhoA-glutathione *S*-transferase (GST) activation assays were performed with GST-Rhotekin-RBD from Cytoskeleton Inc. Bradykinin was purchased from Sigma, and fasudil and Y27632 were purchased from Selleck Chemicals.

### MEC permeability assay.

A gold standard Transwell permeability assay was used to assess ANDV N-protein-directed MEC permeability (31, 32, 53) on Costar Transwell plates (3-µm pores; Corning) in triplicate. FITC-dextran (40 kDa, 0.5 mg/ml; Sigma) was added to the upper chamber, and the lower chamber was monitored for FITC-dextran 1 h later with a BioTek FLx800 fluorimeter (490 nm/530 nm) (31, 32, 53). The fold change in FITC-dextran transit across ANDV N-protein-expressing MECs versus control MECs was determined (31, 32, 53). Where indicated, 2 days after ANDV infection (MOI, 0.5), MECs were grown overnight in growth factor starvation medium and subsequently stimulated with VEGF-A (100 ng/ml) 1 h prior to the addition of FITC-dextran (31, 32, 35, 52). For bradykinin permeability analysis, MECs were mock, ANDV, or HTNV infected (MOI, 1) in Transwell plates in triplicate and at 3 dpi they were incubated in EBM–0.5% BSA for 2 h. MECs were treated with or without fasudil (10 µM) for 1 h and subsequently stimulated with bradykinin (1 µM) in the presence of FITC-dextran (40 kDa) for 30 min prior to fluorimetric quantitation of medium in the lower chambers and comparison to mock-infected controls. Data presented represent results of three independent experiments (*P* < 0.001).

### Immunoprecipitation analysis.

WB assays were performed as previously described (32). Briefly, MECs were infected with ANDV and grown under normoxic or hypoxic conditions as indicated. Cells were lysed in buffer containing 1% NP-40 (150 mM NaCl, 40 mM Tris-Cl, 10% glycerol, 2 mM EDTA, 10 nM sodium fluoride, 2.5 mM sodium pyrophosphate, 2 mM sodium orthovanadate, 10 mM β-glycerophosphate) with protease inhibitor cocktail (Sigma) (114). Total protein levels were determined, and 20 µg of protein was resolved by SDS-polyacrylamide (10%) gel electrophoresis. Coimmunoprecipitations were performed in lysis buffer as previously described (114) with the antibodies indicated overnight, followed by protein A/G agarose, three washes in lysis buffer, and resuspension in SDS sample buffer prior to SDS-gel electrophoresis and WB analysis (32). Proteins were transferred to nitrocellulose, blocked in 2% BSA, incubated with the antibodies indicated, and detected with horseradish peroxidase-conjugated anti-mouse and anti-rabbit secondary antibodies and ECL reagent (Amersham).

### Statistical analysis.

Results were derived from two to five independent experiments and are presented as the mean ± the standard error of the mean (SEM), with indicated *P* values of <0.01 and <0.001 considered to be significant. Multiple group comparisons were made by one-way analysis of variance. Two-way comparisons were performed by two-tailed, impaired Student *t* test. All analyses were performed with GraphPad Prism software version 5.0.
